# Can Circumstances Be Softened? Self-Efficacy, Post-Migratory Stressors, and Mental Health among Refugees

**DOI:** 10.3390/ijerph18041440

**Published:** 2021-02-04

**Authors:** Henriëtte E. van Heemstra, Willem F. Scholte, Angela Nickerson, Paul A. Boelen

**Affiliations:** 1 ARQ Centrum’45, ARQ National Psychotrauma Centre, Nienoord 5, 1112 XE Diemen, The Netherlands; w.f.scholte@amsterdamumc.nl (W.F.S.); p.a.boelen@uu.nl (P.A.B.); 2 Department of Clinical Psychology, Utrecht University, Heidelberglaan 1, 3584 CS Utrecht, The Netherlands; 3 Department of Psychiatry, Amsterdam UMC, University of Amsterdam, Meibergdreef 9, 1105 AZ Amsterdam, The Netherlands; 4 School of Psychology, University of New South Wales, Sydney, NSW 2052, Australia; a.nickerson@unsw.edu.au

**Keywords:** refugees, self-efficacy, post-migratory stressors, mental health problems, non-clinical population

## Abstract

Post-migratory stressors (PS) are a risk factor for mental health problems among resettled refugees. There is a need to identify factors which can reduce this burden. Self-efficacy (SE) is associated with refugees’ mental health. The current study examined whether SE can protect this group from the impact of PS on mental wellbeing. Higher levels of PS were expected to be associated with higher levels of mental health problems. In addition, we expected this linkage to be moderated by lower SE. Questionnaires were administered to a non-clinical refugee sample (*N* = 114, 46% female, average age 35 SD = 10.42 years) with various backgrounds. The following questionnaires were used: the Self-Reporting Questionnaire-20 (SRQ-20) to assess mental health problems, the General Self-Efficacy Scale (SGES) to measure SE, and an adapted version of the Post-Migration Living Difficulties Checklist (PMLD) to measure PS. Bivariate correlations and multiple linear regression analysis were performed. No significant contribution was found for SE or the interaction of SE and daily stressors, above and beyond the significant contribution of daily stressors to mental health problems. The findings reinforce that PS affects mental health and suggest that SE had a limited impact on mental health in this non-clinical sample of refugees.

## 1. Introduction

The worldwide number of refugees has continuously increased since 2005. By the end of 2019, the number of forcibly displaced people was 79.5 million, 26 million of whom were registered as refugees [1]. Forcibly displaced people have left their homes as a consequence of social and political or other events that disorganize public stability [2]. The sub-group of refugees contains people who are defined by the UNCHR as “someone who is unable or unwilling to return to their country of origin owing to a well-founded fear of being persecuted for reasons of race, religion, nationality, membership of a particular social group, or political opinion” ([3], page 3). This group is at high risk of developing mental health problems [4,5,6,7]. To illustrate, the prevalence of common mental health disorders among refugees is about twice as high compared to migrant worker populations [8].

Factors contributing to these mental health problems can be roughly divided into two categories. First, as an increasing number of studies have shown, refugees are exposed to many traumatic experiences, inflating the risk of mental health problems and psychiatric diagnoses, such as posttraumatic stress disorder (PTSD) and depression [9]. The second established factor that threatens the mental wellbeing of refugees is post-migratory stress [10,11,12,13]. Examples of post-migratory stressors are social isolation [14], unemployment [15], and discrimination [16,17].

Although intervention studies among refugees are relatively scarce, trauma-focused interventions have been recommended as first-line interventions for PTSD in refugees [18,19]. Unfortunately, there is less clarity on how to intervene on the profound effects of post-migratory stressors [20]. Evidently, the impact of post-migratory stressors can be partially reduced by practical changes, such as obtaining a job or increased proficiency in the host country language [21], as well as by policies enabling these factors [22]. This level of intervention, however, often requires policy change in host countries, and thus is usually beyond the influence of individual refugees and their helpers. Therefore, increasing personal resources for dealing with these post-migratory stressors is crucial. A focus on resilience building within refugee communities is recommended [23]. However, the psychological mechanisms underlying the association between post-migration stressors and mental health outcomes, that might be targeted to improve resilience, are still largely unclear [12,21].

Self-efficacy, the individual perception of one’s personal ability to deal with upcoming challenges and stressors [24], may be one key mechanism moderating the relationship between post-migratory stress and mental health problems. Previous research among refugees revealed its positive association with mental health and positive post-migratory outcomes (e.g., employment) [25]. Additionally, self-efficacy predicted positive affect over a time period of two years among a group of refugees living in the United Kingdom [26]. An experimental study demonstrated that enhancing self-efficacy led to increased distress tolerance among treatment-seeking refugee torture survivors [27]. Although these studies underline the importance of self-efficacy for refugees, it still needs to be determined whether self-efficacy mitigates the negative impact of post-migratory stressors on mental health problems within this group.

The current study examined the potential moderating role of self-efficacy in the relationship between post-migratory stressors and mental health problems, in a non-clinical sample of refugees residing in the Netherlands. We expected that higher levels of post-migration stressors would be associated with higher levels of mental health problems. In addition, we expected this linkage to be moderated by lower self-efficacy. Findings can be used to guide (preventive) mental health programs and policies for refugees.

## 2. Materials and Methods

### 2.1. Procedure

The current study used a cross-sectional design. Participants were recruited via six different non-governmental organizations (NGOs) operating in the area of Amsterdam, the Netherlands. Measurements were primarily conducted to monitor various support programs offered by these NGOs, focussed on job skills and empowerment. The current study utilized their baseline measurements for secondary data analysis. The aim of their evaluation was to investigate whether their programs were associated with increased empowerment, measured by changes in self-efficacy, and quality of life, measured by post-migration problems and mental health problems. Subsequently, the collected data were deemed suitable for our research objectives.

Participation was voluntary and participants gave informed consent before filling out the questionnaires. Questionnaires were available in Dutch and English. Additionally, the questionnaires were translated from Dutch and/or English (depending on the translators’ preference) into Arabic and Tigrinya, the most prevalent languages in the study sample, using a back-and-forth method, with discrepancies being reconciled. Translators were accredited translators or bilingual individuals with experience in working with refugees.

The self-report questionnaires were administered during group meetings, just before the participants started a group program aimed to increase their personal skills in dealing with work or social challenges connected to their refugee status. The content of these support programs differed between the participating NGOs. It was guaranteed that the data would be anonimized, and participation was voluntarily. Participants were instructed to fill out the questionnaires individually.

Assistance to the participants during the administration of the questionnaires was provided by researchers and/or bilingual professionals who were instructed by the researchers. They also checked questionnaires for missing responses directly after administration and requested the participants to complete missing items when applicable.

The Utrecht University medical ethical review board declared that there was no need for review of the ethical merits of the current study, because the questionnaires were primarily administered for evaluating the NGO programs.

### 2.2. Participants

One hundred and fourteen (*N* = 114) refugees participated in the current study. Their characteristics are listed in Table 1. All participants had a temporary residency permit, which indicates that, in general, they received a legal residency permit less than 5 years ago. The target groups of the participating NGOs overlapped with the inclusion criteria of the current study, which were (1) being a refugee, (2) age ≥ 18, and (3) available informed consent regarding the data collection and analysis. There were no exclusion criteria. Participants had been referred to the NGOs by their personal (online) network, charity organizations, or governmental organisations. 

Because the current study was based on secondary data analysis, no sample size calculation was made prior to the data collection. However, after the current study was designed, an estimation was made to check if the current sample size was satisfactory. A sample of *N* = 114 was found to suffice for detecting a moderation effect, explaining 6.5% of the variance by the interaction effect of self-efficacy and daily stress, in the context of multiple regression, with a power of 0.80.

### 2.3. Questionnaires

#### 2.3.1. Self-Reporting Questionnaire-20

The Self-Reporting Questionnaire-20 (SRQ-20) was used to measure general health problems within the last 30 days. Participants were asked to respond to 20 questions (2-point scale: “*yes*” or “*no*”), regarding their mental health (e.g., “*Do you feel nervous, tense or worried?*”). The questionnaire, developed by the World Health Organisation [28], was validated in several cultural contexts [29,30]. The Cronbach’s alpha in the current study was 0.84.

#### 2.3.2. General Self-Efficacy Scale

Self-efficacy was measured with the General Self-Efficacy Scale (GSES) [31]. Participants were asked to rate 10 items (e.g., “*Thanks to my resourcefulness, I know how to handle unforeseen situations*”) on a 4-point scale (ranging from “*not at all true*” to “*exactly true*”). The internal consistency and multicultural validity of the questionnaire are endorsed [31,32]. Cronbach’s alpha in the current study was good (alpha = 0.81).

#### 2.3.3. Post-Migration Living Difficulties Checklist

Daily stressors were measured with the Post-Migration Living Difficulties Checklist (PMLD) [33]. This questionnaire was adapted to the specific situation and characteristics of the study population, in cooperation with cultural mediators. For example, the item “*little government help with welfare*” was changed into two items, namely “*little help from charities*” and “*little help from the government*” since the target population often experiences these two sources of help as very different. The cultural mediators were people with a refugee background working or volunteering for the participating NGOs. Participants rated the burden they experienced from 11 potential daily stressors (e.g., “*poverty*” and “*communication problems in the Netherlands*”) on a visual analogue scale (VAS) (from 0 = “*not a problem at all*” to 100 = “*a very big problem*”). Cronbach’s alpha in the current study was acceptable (alpha = 0.77).

### 2.4. Statistical Analyses

SPSS version 23.0 was used to perform the statistical analysis. Missing values were avoided as much as possible, as described above. Bivariate correlations were calculated in order to reveal the correlations between self-efficacy, mental health problems, and daily stressors. Hierarchial linear regression analysis was used, following the enter method, to examine the study hypothesis. The predictor variables were mean centered before the analysis was conducted. In the first step, self-efficacy and daily stressors were entered as independent variables. In the second step, the interaction between these variables was added as a predictor to the model. The SRQ-20 (mental health problems) was entered as the dependent variable. Before running the analysis, several assumptions were checked. No outliers were found and the data were distributed normally. Multicollinearity levels indicated enough independence of the different predictors (see Table 3). Listwise deletion was applied for missing items.

## 3. Results

Descriptives are listed in Table 2.

Men scored significantly higher on self-efficacy compared to woman (*p* = 0.046). The scores on mental health were significantly different (*p* = 0.029) between origin groups, with the lowest scores for Eritreans, followed by Syrians, and the highest scores for participants from other countries. They did not differ by age, gender, or background on any other variable in relation to mental health, self-efficacy, and postmigration stressors (*p* > 0.05). Self-efficacy was not significantly correlated with post-migration stressors (r = 0.01) nor general mental health problems (r = −0.07). Post-migration stressors and general mental health problems were significantly positively correlated (r = 0.31, *p* < 0.001).

The results of the regression analysis are summarized in Table 3. In step 1, self-efficacy and PMLD were added to the model as independent variables, and mental health was added as the dependent variable. Adding the predictors to the model resulted in a significant increase in *R*^2^ (F(2, 102) = 4.40, *p* < 0.05), indicating that these variables explain 7.9% of the variance in mental health. In step 2, the interaction between self-efficacy and PMLD was added to the model as an additional independent variable, which did not result in a significant increase in *R*^2^ (F(1, 101) = 2.24, *p* = 0.138). PMLD was the only variable explaining unique variance in mental health. Self-efficacy and the interaction between self-efficacy and PMLD did not contribute to the explained variance in mental health.

## 4. Discussion

The objectives of the current study were to determine (a) the relation between post-migration stressors and mental health, (b) the relation between self-efficacy and mental health, and (c) the moderating role of self-efficacy in the relationship between post-migratory stressors and mental health problems, in a Dutch refugee sample. We expected that higher levels of post-migration stressors would be associated with higher levels of mental health problems. In addition, we expected this linkage to be moderated by lower self-efficacy. The results only partly confirm our expectations.

A first main finding was that post-migration stressors explained significant variance in mental health problems among the study population. This agrees with prior evidence that post-migration stressors are relevant for the psychological wellbeing of refugees [34]. Our findings support prior recommendations [35] that (preventive) mental health interventions and policies should consider the impact of post-migratory problems.

We did not find a significant contribution for self-efficacy to mental health problems, which contradicts our hypothesis and previous findings among refugee populations [25,27]. Additionally, we did not find a significant moderation effect for self-efficacy, which was also contrary to our expectations. Our study is not the first to find that self-efficacy is unsupportive for mental health among refugees. A recent study among refugees resettled in Turkey and Sweden even revealed that self-efficacy was, via emotional suppression, correlated to psychological distress [36], but the link to post-migration stressors was not examined. A literature review [37] suggests that self-efficacy is not exclusively advantageous in relation to stress and mental health. For example, one study among patients with somatic conditions indicated that high self-efficacy combined with limited control over pain was related to elevated mental health problems [38]. A comparable mechanism could explain the absence of a relationship between mental health and self-efficacy in our study. That is, since refugees generally experience high uncontrollability over the daily stressors [39], they may experience a friction between their self-efficacy and actual control over circumstances that impact their lives, which can abolish the supportive role of self-efficacy [25,27]. This assumption could be examined in future research by including socio-political factors that objectify the actual control that individuals have over their environmental stress, next to mental health and self-efficacy.

The current study has several limitations. First, the design was cross sectional and consequently it remains unclear how the examined parameters interact on a longitudinal basis. It would, for example, be valuable to know if prior self-efficacy levels affect the impact of later upcoming stressors on mental health. Secondly, the use of questionnaires for non-western populations, as in the current study, has been criticized [40]. In addition, questionnaires were administered in a group setting, which is a third limitation, since this may have had an impact on the response tendencies of participants [41]. The presence of peers may, for example, result in socially desirable responses. Fourth, the postmigration stress questionnaire items are limited. Although the content was adapted to the specific sample (see Section 2), the items do not represent the entire scope of postmigration problems that refugees in different contexts may experience. Therefore, we should be cautious in drawing conclusions about the impact of other, non-assessed, stressors on mental health. Additionally, the total load of post-migration stressors was included in the analysis, which does not display information on the impact of the separate stressors. Sixth, the items on the postmigration stress questionnaire were administered on a VAS scale, which has not been validated in previous work. The original five-point ordinal scale ranges from “no problem” to “a very serious problem”. Lastly, we had no data about characteristics of people who were unable or unwilling to participate. Therefore, this study could not control for any selection bias. Additionally, to limit the burden for participants, a limited number of questions was administered on demographic features. Consequently, the impact of the precise duration of the refugees’ stay in the Netherlands on the examined mechanisms remains uncertain.

The study also has several strengths. First of all, this study gains insight into mechanisms underlying mental health for refugees. Despite its relevance, this is a relatively under-researched topic [12,21]. Secondly, the study was performed in a naturalistic setting which contributes to the external validity of the findings. Third, attention was paid to the cultural validity of the questionnaires by using translators or bilingual individuals, and using a back-and-forth method, with discrepancies being reconciled. Lastly, all refugees had a temporal recidency permit. Despite the fact that the number of items on the post-migratory stressors questionnaire was limited, the study population was demarcated on this relevant characteristic which impacts their living conditions and possiblities [42].

Considering these strengths and limitations, several remarks must be made. First, we should be cautious with generalizing our conclusions to other groups of displaced populations (e.g., refugees with a permanent recidency permit or asylum seekers). Moreover, all participants were connected to an NGO, and therefore generalizing the current findings to refugees who are less embedded in their host country should be done with caution. Secondly, the amount of administred postmigratory stressors and characteristics was limited. We therefore recommend future research to include a larger sample and expand the items in the data collection, to objectify the relevance of independent stressors and demograpic characteristics. To increase the cultural validity, a multimethod (e.g., qualitative and quantitative) design is advised for future studies. To investigate the longitudinal relevance of our findings, a cohort study would be a suitable next step.

## 5. Conclusions

The findings from this study endorse the relevance of post-migratory stressors for mental health among refugees with relatively low levels of mental health problems. The findings are in line with prior work [34,36], and illustrate the applicability of prior research findings to the situation of refugees in the Netherlands. In addition, this study was, to best of our knowledge, the first to examine a potentially moderating effect of self-efficacy for the relationship between post-migration stressors and mental health in refugees. Its findings shed light on mechanisms underlying the resilience of a vulnerable population. Counterintuitively and in contrast with several other study findings [25,27], no impact of self-efficacy on mental health was found, and neither did it moderate the relationship between daily stressors and mental health. Our findings dissuade preventive inverventions to focus on increasing control over circumstances that may be beyond the influence of an individual, circumstances which may often prevail for refugees, seriously constraining personal control and agency. Considering that the current study focusses on a non-clinical population, findings are relevant to policies directed at tertiary prevention for resettled refugees.

## Figures and Tables

**Table 1:** Participants’ characteristics (*N* = 114).

Variable	*N*	(%)	M	(SD)	Range
Demographic characteristics					
Gender					
Female	52	45.61			
Male	56	49.12			
Missing	6	5.27			
Age in years	98	85.96	35	(10.42)	21–65
Missing	16	14.04			
Background					
Syrian	66	57.9			
Eritrean	12	10.5			
Other background	17	14.9			
Missing	19	16.7			

**Table 2:** Descriptives (*N* = 114).

Variable	N	(%)	M	(SD)	Range
SRQ-20	110	96.49	0.34	(0.23)	0–1.00
GSES	106	92.98	2.94	(0.54)	1.60–4.00
PMLD	109	95.61	34.88	(19.29)	0–100
Worries about housing situation	108	94.74	22.96	(31.53)	0–100
Interaction with roommates	106	92.98	30.41	(37.05)	0–100
Contact with Dutch institutions	105	92.11	33.64	(30.55)	0–100
Little help from government	101	88.59	34.07	(34.89)	0–100
Little help from charities	106	92.98	26.55	(33.40)	0–100
Being separated from family	108	94.74	33.04	(39.42)	0–100
Worries about family back at home	108	94.74	56.82	(38.86)	0–100
Communication in the Netherlands	104	91.22	43.95	(31.51)	0–100
Discrimination	107	93.86	22.81	(28.92)	0–100
Poverty	105	92.11	32.84	(29.86)	0–100
Loneliness and boredom	106	92.98	39.55	(34.78)	0–100

Note. GSES = General Self-Efficacy Scale; PMLD = Post-Migration Living Difficulties Checklist; SRQ-20 = Self-Reporting Questionnaire-20.

**Table 3:** Hierarchical regression analysis on predictors and moderator for general mental health problems.

Predictor	B	SE B	B	*t*	*p*	VIF	Δ*R*^2^	*R* ^2^
Step 1							0.079	0.079
Constant	0.328	0.021		15.519	0.000			
GSES	−0.024	0.040	−0.058	−0.608	0.545	1.00		
PMLD	0.003	0.001	0.276	2.908	0.004	1.00		
Step 2							0.020	0.099
Constant	0.328	0.021		15.623	0.000			
GSES	−0.030	0.040	−0.07	−0.739	0.461	1.01		
PMLD	0.003	0.001	0.291	3.062	0.003	1.01		
GSES * PMLD	−0.003	0.002	−0.143	−0.150	0.138	1.02		

*N* = 105; GSES = General Self-Efficacy Scale; PMLD = Post-Migration Living Difficulties Checklist; GSES * PMLD = interaction GSES and PMLD, VIF = variance inflation factor.

## Data Availability

Restrictions apply to the availability of these data. Data was obtained from the participating NGOs in collaboration with ARQ Centrum’45. The data presented in this study are available on request from the corresponding author with the permission of the third parties.
